# It Is Time to Take Complaints Seriously? An Exploratory Analysis of Communications Sent by Users to a Public Healthcare Agency before, during and after the COVID-19 Pandemic

**DOI:** 10.3390/ijerph21101299

**Published:** 2024-09-28

**Authors:** Claudia Venuleo, Tiziana Marinaci, Camilla Cucugliato, Sonia Giausa

**Affiliations:** 1 Department of Human and Social Sciences, University of Salento, Via di Valesio s.n.c, 73100 Lecce, Italy; tiziana.marinaci@unisalento.it (T.M.); camillacucugliato@gmail.com (C.C.); 2 Local Health Agency (ASL) of Lecce, Via Miglietta 5, 73100 Lecce, Italy; sonia.giausa@asl.lecce.it

**Keywords:** appreciations, complaints, COVID-19, health services, users’ subjective experience

## Abstract

Taking due account of users’ perspectives is crucial for improving the quality of healthcare services. This study aimed to analyse the representations and evaluation criteria that users of a public health agency express towards care and treatment services and to explore whether and how the content and meaning of their communications varied according to pre-pandemic, pandemic or post-pandemic periods. A total of 501 communications sent to the public relations office of an Italian health agency were collected. An automatic content analysis procedure was applied to the textual corpus. Four main thematic cores were identified concerning the request for care and respect, the value of the doctor–patient relationship and the difficulties in contacting services and accessing care. Two main latent dimensions of meaning were identified, which capture the dialectic between the demand for relationships and the demand for access to care, and between attention to the relational competence of health workers and attention to the needs and rights of users. Communications collected during the pre-pandemic and post-pandemic periods mainly concern the difficulty of access to care; those collected during the pandemic period mainly concern the doctor–patient relationship. Interpersonal aspects and timely access to care appear to be crucial in users’ assessment of the quality of care.

## 1. Introduction

There is a growing recognition of the need to embrace patient-centred care (PCC) approaches in the delivery of healthcare, but this centring still seems far from being realized, as indicated by the huge volumes of complaints received by healthcare organizations on a variety of problems related to quality of clinical care, communication, access and other faults during medical practice [1,2]. Although there are no comprehensive international statistics regarding how widespread dissatisfaction is with healthcare encounters, care and treatment [3], national reports and a huge number of studies recognize the extent of patients’ unsatisfaction and the need to take complaints seriously. For instance, data on written complaints made by (or on behalf of) patients in 2021 and 2022 in England [4] show that the total number of all reported written complaints toward primary care and health and community health services was 225,570; this was equivalent to 398.9 complaints per 100,000 head of the population. The Health Complaint Trend Report 2017–2018 to 2021–2022 [2] highlights that there was a 14% increase in total complaints from 2017–2018 to 2021–2022 in Western Australia. The research conducted from February to March 2023 in the U.S. by the Harris Poll on behalf of AAPA [5] shows that more than 70% of U.S. adults feel the healthcare system is failing to meet their needs in at least one way. Patients’ complaints about healthcare encounters are increasingly reported by studies conducted in different countries, such as Switzerland [6], Spain [7], Turkey [8] and Italy [9].

A recent qualitative meta synthesis on twenty-four studies reporting negative patient experiences expressed in complaints identifies four main themes [10]: (1) problems with access to healthcare services; (2) failure to acquire information about diagnosis, treatment and the expected patient role; (3) experiencing inappropriate care and bad treatment; and (4) problems with trusting healthcare service providers. The systematic review of Reader and colleagues [11] on 59 studies, reporting 88,069 patient complaints, distinguishes among three domains, the ‘clinical’ domain which pertains to patient reports on poor quality care and safety incidents; the ‘management’ domain (35.1%) which pertains to problems in waiting times/access to care and institutional management; and the ‘relationship’ domain (29.1%) which considers patient complaints on interactions and experiences with healthcare professionals. A study conducted in Italy—the context of the current study—by Mattarozzi and colleagues [9] adopted Reader and colleagues’ patients’ complaint taxonomy to analyse 1235 instances of complaint and 1536 instances of praise submitted from patients, family members or a legal representative to a northern Italian hospital. The results show that the most frequent causes of complaint concerned care system management (68.1%), particularly the time taken to access treatment, and relationship aspects (52.8%). The most critical factor of the relationship domain was effective communication of information to the patient (39.3%).

It has been widely recognized that putting the patient’s needs at the centre enhances the patient’s experiences of service use and that accommodating preferences means significantly affecting service quality [12,13,14,15]. Previous research highlights how neglecting patients’ perspectives can lead to various negative outcomes: discontinuity of care, compromise of patient safety, patient dissatisfaction and inefficient use of valuable resources, both in unnecessary investigations and physician worktime as well as economic consequence [16]. Conversely, valuing patients’ perspectives can lead to better health outcomes: improved control of the chronic condition, decreased hospitalizations and readmissions [17], improved emotional and physical health status [18], greater satisfaction with the overall quality of care [19,20] and improved quality of the healthcare system [21].

The emphasis on the need to capture patients’ perspectives is also motivated by a different view of the patients: they are no longer identified with their disease and are no longer viewed as passive recipients but recognized as a biopsychosocial unit, characterised by emotions, values, and expectations that inevitably mediate their patterns of use and evaluation of health services [22,23,24,25]. This means that even in the most traditional model of medical practice—where patients are expected to be instructed by clinicians and to follow their suggestions—health outcomes (good and bad) are coproduced [26]. They depend, for instance, on how clinician and patient communicate effectively, develop a shared understanding of the problem and generate a mutually acceptable evaluation and management plan.

On the other hand, the interactions between professionals and patients do not happen in a social vacuum. The healthcare system (its structure and its functions) and the large-scale social forces at work in the wider community support and constrain these interactions; the ability of professionals and patients to work together to co-produce value shifts across time, setting and circumstance [26]. Just think about the COVID-19 outbreak when hospitalized patients and their family members were unable in any way to make decisions about their lives or to be questioned about their needs and experience.

The acknowledgment of the importance of taking due account of the patient’s perspective necessitates that researchers understand how to capture this perspective. Traditionally, user views of healthcare have been collected through patient satisfaction surveys which nevertheless force patients to express themselves within categories defined beforehand by the researcher [9,27]. Furthermore, patients’ satisfactions studies have often reported high levels of satisfaction that do not necessarily reflect a lack of problems or problematic experiences with the healthcare system, but they rather reflect a gratitude or social desirability response bias, enacting the role of the passive patient, as well as beliefs in the legitimacy of their own expectations and lack of willingness to voice discontent [28]. Think again of what happened during the COVID-19 pandemic: although most surveys conducted during this period on patient satisfaction show no significant changes [29], qualitative research highlights how the subjective experience of patients and family members appears to have been significantly impaired [30,31,32]. So, it was suggested that a qualitative approach may be more successful in accessing patients’ views of the quality of care received, since it gives the opportunity for the users to express themselves in their own terms [28,33,34].

Qualitative complaint analysis is increasingly recognised as a unique channel for collecting spontaneous and unsolicited information [8,11,35]. Several authors have recognized the “patient complaint” as a unique and relatively low-cost indicator of the quality of care provided [9,36,37]. Mattarozzi and colleagues [9] emphasize how patients may have a privileged view of issues or problems that healthcare providers fail to recognize or deal with, such as what may occur before a visit or admission, or immediately after; from this perspective, complaints may obviate this blind spot in the process. Similarly, Newdick and Danbury [38] underline how complaint analysis could be particularly important in a system that culturally prevents staff from independently raising issues regarding quality and safety.

This study analyses complaints, appreciations and requests for information sent by patients, family members, or law firms to a public relations service (URP) of a healthcare agency in Southern Italy. The specific task of the URP is to implement, through listening to citizens and internal communication, processes to verify the quality of services and their satisfaction. The URP, thus, provides a privileged context to explore expectations, values and evaluation criteria, that users express with respect to the relationship of assistance and care.

The study was launched in 2023, in the aftermath of the health emergency linked to the COVID-19 pandemic, a period characterised, among other elements, by overworked health workers, a ban on visiting hospitalised relatives, and the exacerbation of organisational problems linked to the lack of material and human resources in places of care and treatment [39,40]. Therefore, it was also considered useful to explore whether and how the contents and meaning of users’ communications had varied according to the pre-pandemic, pandemic and post-pandemic periods; the hypothesis is that the pandemic scenario has confronted new challenges and new problems in the relationship with the healthcare system and therefore made specific content in communication with the URP meaningful.

## 2. Materials and Methods

The study adopted a qualitative approach, based on the collection of written communications—complaints, appreciations and requests for information—received by the URP of a Local Health Agency in Southern Italy. This approach is widely recognized as a unique channel for collecting spontaneous and unsolicited information and to capture users’ perspectives on what has wrong and what has gone right and what information is missing in their subjective experience of the healthcare system [8,11,35].

The study was carried out thanks to the collaboration of the cited URP within the framework of the memorandum of understanding established between the Local Health Agency (ASL) and the Department of Human and Social Sciences of the University of Salento for the implementation of initiatives for training, professional qualification and research in the field of psychology (protocol n.: 111199 of 10 July 2023). The URP collected user communications and shared them with the research team after stripping their sensitive data (e.g., first name, last name, contact, any references to specific persons), respecting the principle of anonymity and confidentiality in accordance with the ASL regulations and information notice to users on the processing of personal data. All procedures were approved by the Ethics Commission for Research in Psychology of the Department of Human and Social Sciences of the University of Salento (protocol n. 144542 of 12 July 2023).

A purposive sampling has been adopted. Consistent with the objective to explore whether and how the contents and meaning of users’ communications had varied according to the pre-pandemic, pandemic, and post-pandemic periods, the URP was asked for all communications received in the following three times, which reflects the temporal evolution of the pandemic in Italy and anti-contagious measures established by the Government:

- September 2019 to February 2020 (pre-pandemic period).
- March 2020 to August 2020 (pandemic period).
- September 2022 to February 2023 (post-pandemic period).

In the first period considered, the health emergency had not yet broken out in Italy and access to hospitals and other healthcare services complied with traditional access regulations. The second period considered coincides with the outbreak of the pandemic, the definition of the nationwide lockdown and important access restrictions in hospitals and other care services (ban on access and/or visits for family members). By the third reporting period, the health emergency had subsided, and the restrictions related to anti-pandemic measures were no longer in place.

The communications were written by patients and family members, sometimes with the assistance of advocacy workers (e.g., from Patients’ Rights Tribunal) and sent to the URP through various channels, such as email, physical desk at the office, portal (from the official website) and via social media (i.e., Facebook page). Communications were eligible for inclusion if, following verification, they had been classified by the investigators as relating to complaints, appreciations towards the health system, or requests for information, consistent with the purpose of the study to capture what does not work, what meets or exceeds expectations and what is the nature of the information that users feel they needed; communications containing requests inconsistent with the function of the URP and not related to experiences with the health system (i.e., reports of lack of public hygiene in the streets or in specific commercial activities, reports of inefficiencies found in private pharmacies, requests for medical records of private facilities, requests for security management during public events, and requests for collaboration on research activities) were excluded. Of the 555 texts collected, 501 were considered eligible for analysis (262 sent by women and 239 by men).

As Table 1 shows, 19.2% of communications were collected in the pre-pandemic period, 38.7% in the pandemic period and 42.1% in the post-pandemic period. In total, 73.4% of the communications were in the nature of complaints, 16% were appreciations and only 10.6% were requests for information; a fact that already signals a certain way of interpreting the function of the URP and how it is used.

## 3. Data Analysis

Traditionally, the handling of users’ communication is carried out on an individual basis. Responses are given on a case-by-case basis and then, generally, the file is archived. However, there is increasing recognition of the importance of analysing this source of information in aggregate to understand the macro- and micro-areas most frequently mentioned by users [36]. This approach also could provide the organization with an insider’s key on user culture both in terms of service use and expectations regarding care and treatment. To this end, an Automatic Procedure for Content Analysis (ACASM; [41,42]), performed using the T-Lab software (version T-Lab 10.2 [43]), was applied to the entire corpus of collected texts, preliminarily stripped of ritual formulas, such as, *cordial greetings*, *cordially*, and *egregious*. ACASM is a kind of semantic analysis sensitive to the contextual nature of linguistic meaning, that is to the assumption that the meaning of any word is not fixed but depends on its combination with other words in the dynamic context of discourse [44,45,46]. Unlike most semantic analysis methods that focus on the co-occurrence of lexical units, ACASM adopts a single sentence or a small group of sentences, called Elementary Context Units (ECUs), as the context unit. Lexical forms within ECUs are categorized according to their corresponding lemmas. Lemmas are the labels that indicate the lexical forms/classes to which a word refers, regardless of its syntactic form [47]. For example, lexical forms “treat”, “treats”, “treated”, and “treating” would be transformed into the single lemma “to treat”. Within this unit, co-occurrences are detected. The multidimensional procedure is applied to the data matrix composed of the segments into which the text is divided (i.e., paragraphs) as rows, lemmas as columns and presence/absence values in cells. Table 2 describes the characteristics of the dataset.

The analysis was performed in two steps. Firstly, the T-LAB software analysis “Thematic Analysis of Elementary Contexts” was performed. It enables the construction of a representation of corpus content through the identification of meaningful thematic clusters [43]. Each cluster is defined by a set of ECUs characterized by the same keyword patterns and is described through the lexical units (lemmas) and variables (in our case, the typology of communication—request for information, complaint or acknowledgement—and temporal block—pre-pandemic, pandemic, or post-pandemic) that most characterize the elementary contexts of which it is composed. The outcome of this analysis is a detailed mapping of general or specific themes [48], highlighting the presence of distinct semantic traits. Interpreting clusters means identifying the thematic core shared by different representational contents conveyed by each grouping. We consider each cluster as the expression of a specific way of representing (of having an opinion, of connoting) the various objects of an experience on which users express themselves. For each cluster, we identify the percentage of texts related to each of the temporal blocks considered (pre-pandemic, pandemic and pot-pandemic) and typology (info, complaints and acknowledgment). Then, in order to investigate the dimensions of meanings which explain similarities and dissimilarities among the texts collected, a Lexical Correspondence Analysis (LCA)—a factor analysis procedure for nominal data [49]—was applied on the contingency table lexical units x clusters [50,51,52], to retrieve the factorial dimensions describing lemmas with higher degrees of association, i.e., occurring together many times.

Broadly speaking, LCA breaks down and reorganizes the relations between lemmas in terms of a multidimensional structure of opposed factorial polarities; where each polarity is characterized by a set of lemmas that tend to co-occur and do not occur in the event of the occurrence of an opposite set. Accordingly, this structure can be interpreted as the operationalization of the semantic structure of the textual corpus under investigation, with any factorial dimension seen as a marker of a dimension of semantic variability. Interpreting each dimension means understanding which common meaning emerges from the subset of lemmas that characterize one polarity and which from the subset of lemmas characterize the other polarity, as well as understanding the second-order meaning that emerges from their aggregation [44]. In the present study, we interpret the first two factor dimensions that correspond to the Latent Dimensions of Meanings that best explain the variability of the data.

It is possible to graphically represent the associations between words and texts on planes bounded by two orthogonal axes. Conventionally, the first factor is represented by the horizontal axis where the negative polarity (−) is placed on the left and the positive (+) polarity on the right, while the second factor is represented by the vertical axis where the negative polarity (−) is placed at the bottom and the positive polarity (+) is placed at the top of the graph. Each factor is associated with a label that identifies a specific mode of symbolization as opposed to another mode, identifying the other pattern.

The variable period of production of the text (pre-pandemic, pandemic, post-pandemic), as well as the nature of the text (complaint, appreciation or request for information) have been included in the analysis as illustrative variables: they, therefore, did not contribute to the definition of factor dimensions; rather, they have been used as a criterion of comparison once their definition has been obtained, to analyse their positioning in symbolic space.

## 4. Results

### 4.1. The Main Semantic Cores

The analysis of the clusters allowed identification of four main semantic cores. Some of the main segments (“typical phrases”) that characterize them are shown in italics. Where reference to names of specific doctors or other professionals or specific departments appeared in the text, the name was blacked out and replaced with ‘xxx’.

Cluster 1: *The right to be cared for and respected*

*“They don’t know where to go and they don’t know how long they have to wait to check the course of their disease. Therefore, the TDM (patients’ court) understands the difficulty of patients, respecting the right to access: every individual has the right to access health services that his or her state of health requires.”*

*“Other waiting patients complained about being greeted with profanity in the background when ringing the doorbell of the ward. I assure you that while it can be irritating for the staff to receive patients, it is certainly not a walk in the park for the patients to come to the hospital in this situation.”*

*“When I went to the ward, I found the patient with the following conditions: open windows with the power going right on the patient; sun beating down on the medications and on the patient; foot bent to the bedside certainly for hours with severe injuries to the foot with related bleeding and sores.”*

*“Every sick person has the right to quality continuity of care, both in and out of the hospital; the right to be welcomed into services by qualified staff who are willing to listen and fully trained as well as the right to have their specificity arising from age and health conditions recognized.”*

*“Do patients remain patients from ward to ward? What happened to the right of a wife, a mother, a child to see a suffering relative in need of 10 min of affection? Does the Covid precaution start from the relatives of patients or from the staff serving in the wards, given the absence of the planned protective device?”*

Cluster 1 collects discourses that refer to the patient’s right to be treated and respected and circumstances in which this right has been lost: for example, waiting lists for access to care that are incompatible with the urgency of the diagnostic question; there are also mentioned situations of neglect towards the hospitalized patient, inadequate communication methods towards patients waiting for an outpatient visit, as well as—with reference to the health emergency—the possibility of family members being close to a suffering relative being denied in the name of a principle of health protection that seems to be denied by health workers without personal protective equipment.

Cluster 2: *Barriers to access to care*

*“Premise: I had been trying to get a visit through CUP (unique reservation centre) for a long time, but since everything was blocked, I decided to find a visit even for a fee. All the facilities contacted told me that the visit was 80 € + 50 for the ultrasound examination. So, all facilities, including xxx, told us that the full visit was 132 €.”*

*“I took my disabled mother-in-law for a visit for ALPI activities with doctor xxx, paid the €82 co-pay regularly and waited in vain in the clinics. After some time, I noticed that no one was there to do the examination, I went to the ward to ask for an explanation and the medical staff told me that the doctor was not at work.”*

*“The prescription for the examination to be done before the visit, I made it back in April as indicated in the attachment, but to date it has not been possible to have the reservation due to the known delays. I have been suggested to have the examination in other hospitals, but I do not have the possibility to go outside xxx.”*

*“I went to the health directorate to complain and after contacting the on-call doctor of xxx ward he/she refused to do the exam stating that ALPI was working in charge of another doctor. I made myself available to regularize the ticket in his name but in vain. After several hours of waiting, I returned at 6 p.m. without performing any examination”.*

*“So, after several checks, a nurse took my prescription and went to the CUP where he was printed the receipt of my reservation, only then I was informed that the doctor’s visit was at another facility. Once I got my car, I went to xxx hospital where I waited for the doctor to call me to make the visit.”*

Cluster 2 collects discourses that refer to the difficulties in accessing assistance: economic difficulties in *undergoing* specialist examinations in the private sector due *to* long waiting times required by the public service, but also difficulties related to inefficiencies (the absence of the doctor during a face-to-face appointment; the vain wait to be received) and unclear or incomplete communications (for example, in place of the appointment).

Cluster 3: *The impossibility of contact*

*“I have called many times, and it is always busy, or rings and they don’t answer. The answering machine picks up, tells me the operator is busy and to call from 9 a.m. to 12 p.m. and two days a week in the afternoon; but I call but will I ever be lucky to get the line, what should I do?”*

*“A week ago, I had called the ASL in xxx, since they were not answering I called the one in xxx, I had talked to an operator and asked if the ASL in xxx should make a reservation to do withdrawals, they answered and told me that I could go without a reservation. Today, however, when I showed up at the ASL of xxx I was told that I had to make a reservation.”*

*“I confirm that no one answers and having contacted the xxx switchboard to get more information; the receptionist confirmed that complaints are widespread and daily because the service staff does not answer the numbers given, despite the fact that it is a paid service.”*

*“I would like to report that the reservation centre is not answering the phone. After two days of continuous phone calls in the afternoon, no one answered. I had to go directly, in a pregnant state, to the counter located on the ground floor of xxx hospital to find out, cell phone in hand, that the phone at the booking point did not make a sound.”*

*“Good morning, I’m txt, I’ve been trying to book the various exams for prevention for days, but at the numbers they gave me and found on the Puglia Salute website no one answers. Can you tell me how we can make reservations? There is a lot of talk about prevention, but it is impossible to make an appointment over the phone”*

Cluster 3 collects discourses that refer to the difficulty/impossibility of getting in touch with operators and services responsible for booking examinations and visits: telephones that are always busy, operators that do not answer, and telephone numbers that are not active.

Cluster 4: *The value of the doctor–patient relationship*

*“Good morning, I am the daughter of Mr. and Mrs. xxx who came to you on the day for a xxx in the emergency room of the xxx presidio. The emergency room promptly assisted dad and mom, thanks also to you. I would like to take this opportunity to thank and express our gratitude for how the team at xxx hospital extended care for my dad. A heartfelt thank you to the head doctor Dr. xxx.”*

*“This is meant to be a praise to Dr. xxx, not to mention the invaluable intervention of the team that performed the operation. All of us, relatives and friends of the patient, wish to express our deep and sincere thanks to the said Doctor, as a doctor with professionalism and great humanity.”*

*“At a sad and desperate time in our lives, I met exceptional people. People who work hard in your hospital and whom you can be proud of. I wanted you to share my thoughts for the doctor who, with great professionalism, contributes prestige. I have sent you an attached letter. I hope it may be appreciated.”*

*“On behalf of myself and my entire family, I want to extend a simple thank you for the dedication and determination shown by all the teams, but in particular an endless thank you to the doctors of the xxx department. I ask that all departments in the hospital be as efficient as xxx’s department is, thank you.”*

*“I wanted you to share my thoughts for the doctor who, with great professionalism, gave his life. I have sent you a letter attached. Thank you from the bottom of my heart. Letter: To the doctor with infinite gratitude. sometimes it is difficult to express in words what is in one’s heart.”*

Cluster 4 collects discourses that refer to gratitude for the care received, emphasizing characteristics such as timeliness, determination, humanity and dedication shown by specific health professionals or entire hospital teams in care of the sick.

### 4.2. The Latent Dimensions of Meaning

Table 3 and Table 4 illustrate, respectively, the first and the second factorial dimensions obtained by the LCA. For each polarity of the two dimensions, the lemmas with the highest level of association (V-Test) are reported, as well as their interpretation in terms of labelling their meaning. Henceforth, we adopt capital letters for labelling the dimensions of meaning and italics for labelling polarities and for indicating the lemmas that characterise them.

#### 4.2.1. First Dimension: Kind of Demand Addressed to the Health System

The first dimension organizes two different types of demand addressed to the health system that can be interpreted in terms of the dialectic: *Demand for relationship* vs. *Demand for access to care* (Table 3)

(−) *Demand for relationship.* On this polarity, lemmas that refer to a dimension of gratitude (*thank you*, *to thank*, *thanksgiving*, *gratitude*), co-occur with lemmas that refer to the doctor–patient relationship, qualifying it, positively or negatively (*violation*) with respect to dimensions of *humanity*, *competence*, professionalism, and with reference to the context of hospitalization (*hospital*, *ward*) and critical health conditions (*illness*, *life*), of oneself as a patient or of a family member (*mom*). On the whole, the polarity seems to refer to the close relationship drawn by the writer between relationship and *care*.

(+) *Demand for access to care.* On this polarity, lemmas referring to procedural dimensions (*booking*, *to book*, *payment*, *prescription*), communication channels (*telephone*, *number*, *email*) and service (*CUP*, *office)* that mediate access to the health system (*visit*, *appointment*, *examination*, *check-up*), co-occur with lemmas that refer to contact by the user to receive answers to their questions (*call*, *ask*, *contact*, *request*, *communicate*, *office*, *to ask*, *ask*, *to answer*).

#### 4.2.2. Second Dimension: Focus of Discourse

The second factor accounts for two different focuses of the discourse: on the one hand, the *competence required of the healthcare provider* and, on the other hand, the *needs and rights of the service user* (Table 4).

(−) *Competence required of the healthcare professional*. On this polarity, lemmas that refer to communication (*call, to contact, office, answer, word*) and to professionalism and competence (*doctor, professionalism, professional, competence*) co-occur with lemmas that refer to specific affective relationships (*mom, dad, son*) and the experience of seeing the patient recognized in his totality and specificity (*person, suffering, particular, to feel, humanity*) with related feelings of gratitude (*thank you, thanksgiving*) or the denial of this experience (*violation*), as when the perceived experience is of being treated as a number.

(+) *Needs and rights of the service user*. On this polarity, lemmas that refer to different phases of hospital assistance and intervention (*to access, intervention, first aid, assistance, to recover, surgery, hospitalized, therapeutic, drug*), qualified in terms of *quality* parameters, co-occur with lemmas that recognize the user of the health system (*health service, system, health*) both as a patient in need of assistance and treatment (*patient, hospitalized, affection, pathology, serious*) and as a *citizen* with *rights*.

**Figure 1:**
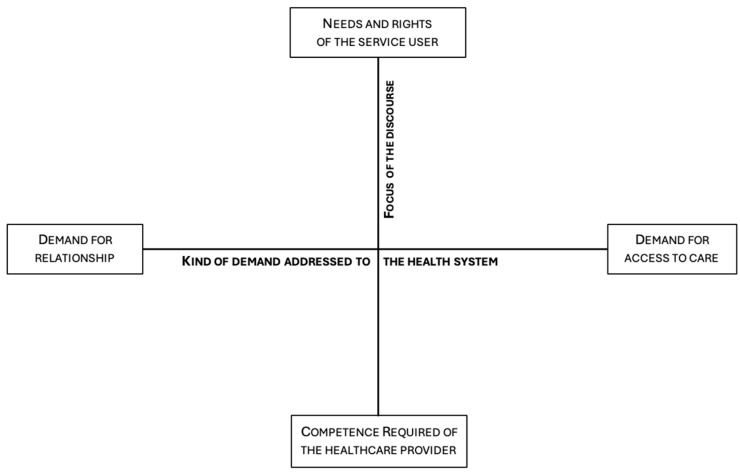
The symbolic space defined by the two main latent dimensions of meaning.

Figure 1 describes the symbolic space bounded by the two factorial dimensions described above.

### 4.3. Relationships between Dimensions of Meaning and Texts’ Characteristics

Both the period of receipt of the communications and their typology (complaints, appreciations and requests for information) show a different association with the first factorial dimension (*Kind of demand addressed to the health system*) obtained by the LCA (Table 5). More particularly, appreciations and texts collected during the pre-pandemic and pandemic periods—months characterized by the overload of health systems, and by anti-contagious measures that prohibited or severely limited the access of family members to visits—tend to be placed on the negative polarity (*Request for relationship*), organized by the recognition of a close connection between treatment of disease and care of relationships. On the other hand, complaints and requests for information texts collected in the post-pandemic period tend to be placed on the positive polarity (*Demand for access to care*), organized by the demand for access to care and the procedural and organizational difficulties that hinder the satisfaction of this demand.

The period of production of the texts and their nature present specific associations with respect to the polarities of the second factorial dimension (*Focus of the discourse*) (Table 5). Appreciations, requests for information and texts produced in the pre-pandemic period tend to be placed on the lower polarity (*Competence required of the healthcare provider*), where the client’s demand is to be supported, in his/her treatment pathway, both from the point of view of technical competence and from a relational and emotional point of view. On the contrary, complaints and texts produced in the post-pandemic period tend to be placed on the higher polarity (*Needs and rights of the service user e*), where the client’s demand is to be recognized not only as a patient suffering from a pathology and therefore in need of care, but also as a citizen, with specific rights of assistance and care.

As Table 6 shows, the clusters present specific associations with the two factorial dimensions identified. More particularly, along the first dimension, cluster 1 “The right to be treated and respected” and cluster 4 “The value of the doctor–patient relationship” relate to the demand for relationship polarity, while cluster 2 “Barriers to access to care” and cluster 3 “The impossibility of contact” relate to the opposite polarity *Demand for access to care.* With regard to the second factorial dimension, only cluster 3 “The impossibility of contact” relates to the negative polarity (Competence required of the healthcare provider); the other clusters relate to the positive polarity *(Needs and rights of the service user*).

## 5. Discussion

The study aimed to explore the thematic contents and evaluation criteria characterizing the communications sent by citizens to the URP of a Local Health Agency in Southern Italy, and captured what went wrong and what went right in the subjective experience of the users. The findings highlight the importance of both interpersonal skills in service delivery and organisational aspects that allow access to care at the right time.

Cluster analysis led to the identification of four semantic cores. It is interesting to note that two of them focus on relational competence skills required from those working in the healthcare system. Attention to the relationship is both asserted as a right, which users claim when they feel they have been treated insensitively and/or have been poorly listened to (cluster 1 “The right to be cared for and respected”), and recognized as a value, qualifying the service received, when empathy, attention, humanity were perceived in the operator (cluster 4 “The value of the doctor–patient relationship”). The other two semantic cores focus on more organisational aspects, which constrain and, in some cases, prevent the very use of services (cluster 3 “The impossibility of contact” and cluster 2 “Barriers to access to care”).

The URP (to whom communications are addressed), therefore, on the one hand is called upon to act as a guardian and guarantor of crucial aspects for the smooth functioning of the service, which sometimes seem to be missing or faltering, such as the right to be cared for, to receive answers, and to have empathetic and respectful relationships with professionals, and on the other hand, is to mediate communications of appreciation when these same aspects of functioning of the health system seem to be working well.

Regarding the results of the LCA, findings show how the texts collected are guided by two fundamental dimensions of meaning. One concerns the kind of demand addressed to the health system and can be described in terms of the dialectic between *Demand for relationship* and *Demand for access to care*.

The *Demand for relationship* polarity—which mainly characterizes appreciations, texts collected during the pre-pandemic and pandemic periods and texts falling within Cluster 1 “The right to be treated and respected” and Cluster 4 “The value of the doctor–patient relationship”—brings to the foreground the value of being treated with respect, empathy, compassion, humanity, to be listened to, welcomed and have their concerns acknowledged. In this case, the discourses focus on the ability or inability of healthcare personnel to meet the standards expected of a formal social encounter (e.g., showing up or not showing up), the time given for the encounter with the patient, the use of sensitive or offensive verbal and non-verbal communication, the willingness or unwillingness to answer questions posed by the user, and the empathy or emotional distance shown towards the patient’s needs and concerns. Users’ communications, thus, invite us to consider that practitioners’ knowledge and technical skills constitute only one side of the healthcare role, which must include compassion, kindness, listening, recognizing the user not just as a patient with a disease but as a biopsychosocial being, bearer of fears, anxieties, and concerns [53]. An analysis of the literature shows how unsatisfactory communication or failed signs of respect and empathic staff responses are common. Hogg and colleagues [54] report that each year with NHS Scotland and associated contractors such as general practitioners, the most commonly mentioned problems are communication issues (47%) and staff attitudes and behaviour (42%), followed by medical treatment (35%). van Mook and colleagues [55], in a study of unprofessional behaviour in healthcare, highlight how complaints about relational aspects of professionalism (such as attention, empathy, communication skills) far outnumber complaints about specific technical and cognitive skills, such as medical mistakes. Complaints about unsatisfactory communication focused mainly on “insufficient clarification/unclear information”. This included lack of clarity in explanations of diagnosis and treatment, often due to the excessive use of medical jargon and failure to provide written information materials, such as pamphlets, to patients. Taylor, Wolfe, and Cameron [56] reported that 32% of complaints about an Australian emergency department related to communication including poor staff attitude, discourtesy and rudeness. It has been observed how within a biomedical approach, focused on the diagnosis and treatment of illness, caring for the emotional aspects of the doctor–patient relationship is often not considered an integral part of the professional role [57]. On the contrary, stereotypes and professional implicit norms often foster health providers’ emotional detachment [58,59], with the view that emotional involvement is an obstacle to care and, thus, unprofessional and inconvenient [60,61]. However, unrecognized emotions in the healthcare providers’ experience may prevent the adoption of a patient-centred style of care and may be associated with harmful behaviours, such as neglecting patients’ psychological issues [62]. Research shows how failure to take care of the relationship affects patient outcomes in terms of their feelings and their confidence in the outcomes of current and future healthcare encounters [54]. On the opposite, being treated with dignity is linked to higher patient therapy adherence and satisfaction, including for end-of-life care [63].

The *Demand for access to care* polarity—which mainly characterizes complaints, requests for information, texts collected during the post-pandemic period and texts falling within cluster 2 “Barriers to access to care” and cluster 3 “The impossibility of contact”—brings to the foreground the need to overcome structural barriers to health. Complaints mainly relate to not being able to get in touch with health services and not having access to healthcare or aids, due to operators not answering the phone and biblical waiting times to get a medical examination. As suggested by Santana and colleagues [64], healthcare is ‘appropriate’ not only when it is cost effective but also when it is provided in the right environment and at the right time. Characteristics of health resources play a central role in facilitating or impeding the use of services by potential users. Policies and organisational aspects of care should be targeted to improve access, which is determined by factors such as the availability, price and quality of health resources, goods and services [65]. Long waiting times for elective procedures are already recognized as a major problem and one of the most important health policy concerns in most OECD countries [66]. In Italy—the context of the current study—excessive waiting lists have been widely documented by independent and National agencies [67,68], especially in the southern regions of the country [69]. Long waiting times in turn inhibit the demand of citizens for public health services and foster inequities in accessing care since the ability to turn to private healthcare, paying additional fees, increases with socioeconomic status [70,71]. Research has suggested that even countries with a universal and egalitarian public healthcare system, like Italy, exhibit a certain degree of SES-related horizontal inequity in health services utilization [72]. Glorioso and Subramanian [72], in a study focused on Italy, found a significant amount of pro-rich inequity in the utilization of specialist care, diagnostic services, and basic medical tests. It is worth noting that excessive waiting time for care and clients’ disinformation about the reasons for waiting has been documented as a key factor for kindling violence against health workers [73]. Previous studies, including patients with serious illnesses, often report similar issues related to poor communication, disorganized services, and long waiting times. These factors remain significant barriers to care and undermine patient-centred approaches, regardless of the severity of the condition [74]. Consequently, organizational inefficiencies and inadequate communication can profoundly affect patients’ care experiences and increase their likelihood of filing complaints, whether they are dealing with a chronic condition or an acute illness.

The second factorial dimension highlights how users’ communications focus alternately on the *Competence required of the healthcare provider* and on the *Needs and rights of the service user*. The first polarity—which mainly characterizes appreciations, requests for information, texts collected during the pre-pandemic period ad texts falling within cluster 3 “The impossibility of contact” and cluster 4 “The value of the doctor–patient relationship”—puts emphasis on the value of patient-centred care and treatment; this approach implies both attention to responding to the patient’s needs within a lawful timeframe and attention in order to not make the user feel like a number, to respect their dignity, to listen to their needs, and to treat them with kindness and sensitivity. The other polarity—*Needs and rights of the service user*—which mainly characterizes complaints, texts collected during the post-pandemic period and texts falling within cluster 1 “The right to be treated and respected”, identifies a different area of meaning, where the patient’s need for timely clinical investigation, examination, and specific treatment, is asserted as a right. Critical medical conditions and suffering are evoked, where sometimes the patient’s life is at stake and where, therefore, any inefficiency in responding to their needs is particularly critical. Communications, sometimes written by the Tribunal for Patients’ Rights, to which the patient or a family member has turned to, to complain about a lack of care, often refer to Article 32 of the Italian Constitution, according to which the Italian healthcare system is built on a universalistic concept of solidarity and promises to ensure care and assistance to all, regardless of nationality, residence, and income. Other times reference is made to the Hippocratic Oath, which in its modern version dating back to the 21st century A.D., refers to principles such as the doctor’s duty to pursue the defence of life, the protection of physical and mental health, the treatment of pain and the relief of suffering with respect to the dignity and freedom of the person. The long waiting times to receive the requested service, in the face of the urgency with which the user is confronted, together with the economic impossibility of turning to a private party, are perceived and presented as a violation of these principles and rights of the citizens.

It is interesting to observe how appreciations and communications received during the pre-pandemic and pandemic periods tend to be characterised by an emphasis on the relational aspects of care. In contrast, complaints and communications received during the post-pandemic period concern more organisational and structural aspects. It is possible to hypothesise that being treated with humanity, kindness, and compassion is not taken for granted in the experience of users, so that when they are confronted with relationally competent operators, they feel they have received more than they expected. This is all the more understandable during the pandemic period. Previous studies have highlighted the idealization of the figure of health workers that took place during the pandemic [75], in recognition of the effort made to save lives in a tragic historical period and with limited resources received to cope with the battle. On the other hand, the COVID-19 scenario, characterised—among other elements—by important measures restricting family visits to hospitalised patients, has made the need and demand for relationally competent health workers more cogent. As suggested by Morley and colleagues [76], the challenge for health and social workers was to temper the potentially dehumanising scenario.

Complaints occur when users feel denied of the minimum levels of care and the right to care, due to the difficulty of getting in touch with the health system to book a medical examination or perform a diagnostic test; it is not surprising that such complaints mainly characterize the post-pandemic period. In Italy, the context of the current study, the COVID-19 health emergency happened within a health system already suffering from a progressive decrease in resources allocated for health-related research and public health, characterized by insufficient availability of medical personnel, products, and physical structures. Lockdown measures and access restrictions in hospitals have delayed the demand for diagnostic tests and access to treatment by non-COVID-19 patients, further lengthening waiting lists in the post-pandemic period [77].

A last comment on the interpretation of the function of the URP, as emerged by the typology of the communication sent by the users: in some cases, the URP is used as a channel of appreciations—where timely and relationally competent assistance has been recognized; in other cases, the majority of times, it is used as a receptacle for complaints, frustrations, and claims for rights of assistance that have been perceived to be violated. From this perspective, the major function of the URP recognized by the users seems to be to exercise a control function with respect to the inappropriate behaviour of those who work in the health system (not only health workers, but also those who work at the counter, at reception and booking services) and to guarantee the right to be treated promptly by removing the obstacles (e.g., long waiting lists, booking difficulties, inadequate medical examinations) that deny this right.

### 5.1. Practical Implications

There is a growing recognition of the need to embrace patient-centred care approaches in the delivery of healthcare but there is less agreement on how to make these approaches a reality in everyday clinical practice [78]. Embracing the patient’s experience of care in its complexity is a necessary step in this direction. Complaints and appreciations inform us of patients’ expectations of how a service should work, what aspects they need to be informed about, what they expect from their relationship with a provider, and what they need to be comforted about. Taking this information seriously means that health systems have a unique opportunity to improve patient satisfaction and quality of care. It is worth underlining how welcoming the patients’ point of view does not mean responding to the manifest problem and closing a bureaucratic issue but recognising their communications as a signal of what matters to users, hence the criteria they use to evaluate their experience and relationship with the service. Complaints and appreciations offer a “window of opportunity” to improve health services [79], giving insights into aspects of healthcare that traditional quality and safety reporting systems fail to capture [11,80]. Furthermore, they can help healthcare professionals reflect on their practices.

In recounting above, all their positive experiences with the healthcare system, users emphasise the importance of communication skills, respect for patient views, and empathy, which builds mutual trust, affects information processing and people’s health choices, as highlight by previous research [81]. This finding highlights well how the relational competence of a health worker is a central criterion in the evaluation of the experience with the health system and how there is no technical competence that can compensate for the user’s experience of not being recognized in his or her dignity and being treated with respect. Many complaints could be avoided by equipping healthcare providers with psychosocial abilities, which should become an integral aim of medical education [53]. An ethic of care would involve a health professional being sympathetically understanding of the experience of patients and family members, sensitive and responsive to their emotional and knowledge needs, and engaged to prevent and relieve suffering throughout an illness experience and until the end of life [46,82]. Effective communication requires skills and simultaneously the constant effort to understand what concerns the patient, to convey the message that his/her concerns and feelings are understandable, and the commitment to give oneself time to listen and contain them [83]. As Fenton and colleagues suggested [84], patient satisfaction can be maintained also in the absence of request fulfilment if physicians address patient concerns in a patient-centred way. It is also recognized that the disclosure of information on medical errors reduces dissatisfaction with healthcare encounters and communication [85]. Therefore, it is important also to develop communication plans and strategies to handle patient complaints [86]. Tailored training initiatives in self-awareness and communications skills to convey empathy through facial expressions, gestures, posture, and de-escalation techniques could be useful to this end.

However, the relational competence of health workers is not the only domain made relevant by user communications. Many complaints focus on the very denial of the right of access to care, signalled by very long waiting lists and the absence of answers to urgent care needs. Limited economic resources and personnel also represent important sources of physical and mental fatigue, stress, anxiety, and burnout among health workers [39,87,88], and these components compromise the healthcare workers capacity to provide respectful and compassionate care and to assure effective communication with patients and family members. So, it is equally important to emphasize the need for adequate institutional responses. People’s vulnerability and health workers’ capacity to assure appropriate responses are also constructed by economic and political conditions.

### 5.2. Limitations and Future Research Directions

The results of the present study should be considered in light of some methodological limitations. First, the results cannot be generalized and have to be related to the specific socio-cultural context under analysis. Because evaluation criteria and meanings through which people interpret quality service also depend on sociohistorical conditions and are placed within the sphere of social discourses, we might suppose that, in other Local Health Agency and countries, complaints and appreciations focus on different components of the health system. Consider, for example, how in Italy there is a considerable north–south divide in the quality of healthcare facilities and services provided to the population. It is possible that the focus on aspects related to the right of access to care would have been less pregnant if we had analysed communications addressed to the Public Relations Office of another Local Health Agency located in Northern Italy. On the other hand, it is plausible that the main discourse domains in which the communications’ focus are common to users in different countries, reflecting a more general demand for patient-centred care, and that local specificities impact the frequency and not the content of the domains. For the future, it could be very interesting to compare the communications received from health agencies in different geographical areas and/or differently characterised in terms of human and economic resources.

Another limitation of the research was related to the fact that communications provided to the research team were stripped of sensitive data and socio-demographic information. Therefore, it was not possible to explore whether and in what direction the nature of the communications (appreciations, complaints, requests for information) were associated with variables such as age, socio-economicus status and education level. Previous studies suggest that these factors relate to variations in self-reported satisfaction, which in turn could be related to different patient expectations and perceptions of care [89]. Different expectations could affect the likelihood of expressing complaints or appreciations and the nature of the subject matter of such communications. Another aspect that deserves further investigation is the role of the patient’s health condition and associated life expectancy which could be related to the urgency of having one’s care needs met and complaining about the lack of response. The recent review of Ferreira and colleagues [90] suggests that perceived health status is one of the factors that has the greatest influence on patient satisfaction; however, to the best of our knowledge, few studies have explored whether lower satisfaction also translates into greater willingness to communicate one’s dissatisfaction. Future research should explore this aspect in depth.

## Figures and Tables

**Table 1:** Characteristics of the texts.

Illustrative Variable	Modality	Percentage Value
Period	Pre-pandemic	19.2%
	Pandemic	38.7%
	Post-pandemic	42.1%
Typology	Complaints	73.4%
	Appreciations	16%
	Requests for information	10.6%

**Table 2:** Dataset.

	*N*
Texts in the corpus	501
Elementary contexts (EC)	1787
Types	9411
Lemma	4779
Occurrences (Tokens)	78.511
Threshold of lemma selection	15
Lemmas in analysis	478

Note—Texts in the corpus: number of answers to the open question (corresponding to the number of participants) inserted in the text analysis. Elementary context: sections of text (e.g., sentences, paragraphs, or short texts) characterized by the same keyword patterns. Types: total number of words (i.e., including all linguistic forms) contained in the general corpus. Lemmas: words transformed into headword. Occurrences (Tokens): frequencies of a single lexical unit. Threshold of lemma selection: the value selected to include the lemma in the analysis. Lemmas in analysis: number of headwords inserted in the analysis.

**Table 3:** First factor dimension: Kind of demand addressed to the health system.

(−) Demand for Relationship		(+) Demand for Access to Care	
Lemmas	Test Value	Lemmas	Test Value
Professionality	−11.9054	To answer	9.8461
Thank you	−11.0784	Booking	9.6795
To thank	−10.7237	To book	9.436
Person	−9.0203	Visit	9.0178
Thanksgiving	−8.4761	To make	8.0087
Ward	−8.3159	Request	7.833
Doctor	−8.2677	CUP	7.5992
Patient	−8.1822	Call	7.4565
Care	−7.9482	Examination	7.21
Humanity	−7.7828	Answer	6.9294
Staff	−7.6903	Number	6.3859
Competence	−7.038	Ask	6.1528
Work	−7.0374	To go	6.1185
Violation	−7.0241	Office	6.0547
Word	−6.7799	Prescription	5.8849
Life	−6.6419	E-mail	5.8515
Professional	−6.6229	To contact	5.8034
Demonstrate	−6.4319	To result	5.3581
Particular	−6.2429	Appointment	5.323
Hospital	−6.2197	Communicate	5.1468
Human	−6.2044	Check-up	4.9324
Express	−6.1271	Payment	4.5119
Gratitude	−6.0287	Telephone	4.4783
Illness	−5.9415	To attach	4.2715
Mom	−5.8973	Information	4.2401

**Table 4:** Second factor dimension: Focus of the discourse.

(−) Competence Required of the Healthcare Provider		(+) Needs and Rights of the service user	
Lemmas	Test Value	Lemmas	Test Value
Doctor	−7.0624	Patient	12.3807
Professionalism	−7.0382	Right	11.2772
Thank you	−6.6927	Surgery	8.661
Answer	−6.46	Hospitalized	8.4639
Big	−4.893	To access	8.3802
Thanksgiving	−4.7954	Pathology	7.2226
To feel	−4.7358	Sanitary	6.8835
Humanity	−4.5016	To recover	6.2835
Call	−4.1497	Behaviour	6.2189
Mom	−4.1363	Health	5.6436
Professional	−4.0924	Citizen	5.6425
Answer	−3.9777	System	5.5788
Violation	−3.9166	Drug	5.4057
Particular	−3.8359	Therapeutic	5.1719
Word	−3.7611	To intervene	5.062
Person	−3.7391	Health Service	4.9925
Number	−3.7365	First-aid	4.9666
Dad	−3.5983	Affection	4.7452
To result	−3.4951	Ambulance	4.6434
To demonstrate	−3.3877	To submit	4.4277
Office	−3.3528	Serious	4.3524
Son	−3.2091	To die	4.2613
To contact	−3.2023	Assistance	4.2423
Competence	−3-1427	Quality	4.212
Suffering	−3.0039	To suffer	4.2101

**Table 5:** Association factorial dimensions * temporal periods and typology of communication.

	Factorial Dimension 1: Demand for Relationship (−) vs. Demand for Access to Care (+)	Factorial Dimension 2: Competence Required of the Healthcare Provider vs. Needs and Rights of the Service User
Temporal Periods	Test Value *	Test Value *
Pre-pandemic	−5.68	−4.32
Pandemic	−12.53	-
Post-pandemic	12.93	6.44
Typology		
Complaints	14.41	5.82
Appreciations	−90.8	−24.65
Requests for information	22.67	4.83

* Z-score.

**Table 6:** Association factorial dimensions * clusters.

	Factorial Dimension 1: Demand for Relationship (−) vs. Demand for Access to Care (+)	Factorial Dimension 2: Competence Required of the Healthcare Provider vs. Needs and Rights of the Service User
Clusters	Test Value *	Test Value *
Cluster 1—*The right to be cared* *for and respected*	−21.85	48.50
Cluster 2—*Barriers to* *access to care*	34.02	3.11
Cluster 3—*The impossibility* *of contact*	38.93	−18.82
Cluster 4—*The value of the doctor–patient relationship*	−60.86	−27.65

* Z-score.

## Data Availability

Data will be made available on request.
